# Outcomes of Extracorporeal Membrane Oxygenation (ECMO) for COVID-19 Patients: A Multi-Institutional Analysis

**DOI:** 10.3390/vaccines11010108

**Published:** 2023-01-01

**Authors:** Maged Makhoul, Eitan Keizman, Uri Carmi, Ori Galante, Eduard Ilgiyaev, Moshe Matan, Artur Słomka, Sigal Sviri, Arieh Eden, Arie Soroksky, Danny Fink, Leonid Sternik, Gil Bolotin, Roberto Lorusso, Yigal Kassif

**Affiliations:** 1 Department of Cardiac Surgery, Rambam Medical Center, Haifa 3525408, Israel; 2 Department of Cardiac Surgery, The Leviev Cardiothoracic and Vascular Center, Sheba Medical Center, Tel Aviv 6423906, Israel; 3 Division of Anesthesia, Pain and Intensive Care, Tel Aviv Sourasky Medical Center, Tel Aviv 6423906, Israel; 4 Medical Intensive Care Unit, Faculty of Health Ben Gurion University, Soroka Medical Center, Beer-Sheva 8400711, Israel; 5 Intensive Care Unit, Shamir Medical Center, Zerifin 703301, Israel; 6 Intensive Care Unit, The Baruch Padeh Medical Center, Poriya 1528001, Israel; 7 Department of Pathophysiology, Nicolaus Copernicus University in Toruń, Ludwik Rydygier CollegiumMedicum, 85-094 Bydgoszcz, Poland; 8 Medical Intensive Care, Hadassah-Hebrew University Medical Center, Jerusalem 91120, Israel; 9 Department of Anesthesiology Critical Care and Pain Medicine, Carmel Lady Davis Medical Center, Haifa 3436212, Israel; 10 Intensive Care Unit, E. Wolfson Medical Center, Tel Aviv 6423906, Israel; 11 Intensive Care Unit, Shaare Zedek Medical Center, Jerusalem 91120, Israel; 12 Cardiovascular Research Institute, Maastricht (CARIM), 6200 Maastricht, The Netherlands

**Keywords:** ECMO, COVID-19, mechanical ventilation

## Abstract

Background: In March 2020, COVID-19 was announced as a global pandemic. The first COVID-19 patient was connected to an ECMO device in Israel during that time. Since then, over 200 patients have required ECMO support due to COVID-19 infection. The present study is a multi-institutional analysis of all COVID-19 patients requiring veno-venous (VV) ECMO in Israel. The aim was to characterize and compare the survivors and deceased patients as well as establish risk factors for mortality. Methods: This retrospective multi-institutional study was conducted from March 2020 to March 2021 in eleven of twelve ECMO centers operating in Israel. All COVID-19 patients on VV ECMO support were included in the cohort. The patients were analyzed based on their comorbidities, procedural data, adverse event on ECMO, and outcomes. Univariate and multivariate analyses were used to compare the deceased and the surviving patients. Results: The study included 197 patients, of which 150 (76%) were males, and the mean age was 50.7 ± 12 years. Overall mortality was 106 (54%). Compared with the deceased subjects, survivors were significantly younger (48 ± 11 vs. 53 ± 12 years), suffered less from ischemic heart disease (IHD) (3% vs. 12%), and were ventilated for a significantly shorter period (≤4 days) prior to cannulation (77% vs. 63%). Patients in the deceased group experienced more kidney failure and sepsis. Rates of other complications were comparable between groups. Conclusions: Based on this study, we conclude that early cannulation (≤4 days) of younger patients (≤55 years) may improve overall survival and that a history of IHD might indicate a reduced prognosis.

## 1. Introduction

Coronavirus disease 2019 (COVID-19), caused by Severe Respiratory Syndrome Coronavirus 2 (SARS-CoV-2), was announced as a global pandemic in March 2020 by the World Health Organization (WHO) [1]. At the same time, on 20 March, the first COVID-19 patient was connected to veno-venous extracorporeal membrane oxygenation (VV-ECMO) machine in Israel. Altogether, throughout the first “COVID-19 year”, 211 patients in Israel required ECMO support due to COVID-19-induced respiratory and or cardiac failure.

Even though more than one year has passed since the beginning of the pandemic, the data regarding COVID-19 patients on ECMO are still sporadic, and the knowledge of how to manage these patients is inconclusive [2,3,4,5]. Medical teams are facing daily, unprecedent decision-making situations concerning ECMO support for COVID-19 patients, such as the right timing for cannulation, on-ECMO management, and weaning strategy. In light of the rise in new viral variants worldwide, there is a growing concern about having to deal with ECMO patients again.

We have conducted a nationwide multi-institutional study including all patients with COVID-19-induced respiratory failure requiring ECMO support. This study aimed to characterize COVID-19 patients on ECMO by comparing the deceased and survivors to understand their clinical course better, establish risk factors for mortality, and possibly provide some insights which may be valuable in the management of future COVID-19 patients.

## 2. Materials and Methods

Twenty-one major hospitals provide advanced healthcare in Israel, of which twelve include ECMO service. This is a retrospective multi-institutional study conducted from 1 March 2020 to 31 March 2021, including eleven out of the twelve ECMO centers in Israel. The institutional review board approved the study. The cohort included patients who required ECMO support because of refractory respiratory failure due to SARS-CoV-2 infection. Only patients requiring VV-ECMO were included in the analysis. Veno-arterial (VA) ECMO patients were excluded.

Formally, dedicated teams coordinated the use of ECMO in each of the involved centers separately. These teams relied on the Extracorporeal Life Support Organization (ELSO) guidelines [6], which were accepted by the Israeli ECMO Society (iECMOs). Generally, given PaO_2_/FiO_2_ < 150 with PaO_2_/FiO_2_ < 60 for >6 h or, PaO_2_/FiO_2_ < 50 for >3 h or pH < 7.2 + PaCO_2_ > 80 mmHg for >6 h, in the absence of an absolute contraindication for ECMO, were the primary indicators for ECMO use. When PaO_2_/FiO_2_ ≥ 150, a pH < 7.2 + PaCO_2_ > 80 mmHg for >6 h indicated the need for ECMO. VA-ECMO was applied for hemodynamically unstable patients with echocardiographic signs of reduced left ventricular ejection fraction. All patients had a multi-disciplinary team prior to ECMO support. As a rule, absolute contraindications for ECMO in COVID-19 patients included: active bleeding, advanced active co-existing illness (e.g., oncological disease, dialysis patients, etc.), severe brain damage, and prolonged cardiopulmonary resuscitation, while relative contraindications included: advanced age, multi-organ failure, morbid obesity, and prolonged mechanical ventilation. It is worth mentioning that although prolonged mechanical ventilation was reported as a relevant contra-indication for ECMO, the teams in each center had the final discussion on whether to ECMO the patients or not. For that reason, some patients submitted to prolonged-ventilation were still considered potential candidates and finally supported by ECMO and have been further analyzed, which may generate a potential selection bias.

The above-mentioned criteria for ECMO were applied in periods of the conventional capacity of each center. However, as probably occurred in most countries around the world, the availability of ECMO devicesand the ability to hospitalize and treat ECMO patients varied throughout the year. Thus, each center’s ECMO team was advised to triage candidates for ECMO. This triage was also based on the ELSO recommendations and recognized four states of capacity: normal capacity (patient selection according to the above criteria), contingency capacity tier 1 (extended capacity favoring younger patients and patients with single organ failure), contingency capacity tier 2 (saturated capacity with restrictive criteria and no VA ECMO use) and, crisis capacity (overwhelmed capacity and unavailable ECMO). Being a relatively small country with only twelve centers offering ECMO services, the iECMOs also used this attitude nationally in Israel. Thus, a national iECMOs “headquarters” was instituted to regulate and coordinate patients’ transfers between centers to allow a critical patient to be treated in “higher capacity” centers [7,8].

Regarding procedural matters, cannulation was achieved percutaneously in all cases via jugular vein and/or femoral veins cannulation by the Seldinger technique guided by transthoracic echocardiography (TTE). In cases where TTE did not provide adequate visualization of the cava and the atria, transesophageal echocardiography (TEE) was used to optimize cardiac imaging.

Once under ECMO support, the ventilation strategy was modified to protective lung management, maintaining: low plateau pressure (<25 cmH_2_O), a relatively low fraction of inspired oxygen (FiO_2_ < 50%) to maintain permissive hypoxemia (SpO_2_ ≥ 85%, PaO_2_ 60–80 cmH_2_O), low tidal volume (Vt < 6 mL/kg), the respiratory rate between 4 and 10 breaths for a minute and higher positive end expiratory pressure (PEEP) titration (8–15 cmH_2_O) accordingly. Whenever possible, the use of muscle relaxants was ceased, and sedation decreased to the minimum to achieve spontaneous ventilation and early mobilization of the patients. Hemodynamically, noradrenaline was the first choice for patients requiring vasopressor support. Further hemodynamic support was achieved by vasopressin as the second line and adrenaline as the third. No specific protocol was maintained for hemodynamic stabilization. Unstable patients, despite significant ionotropic and vasopressor support, were converted to VA-ECMO support by adding an arterial cannula, which was performed percutaneously.

Patients needing VV-ECMO for ARDS will invariably receive mechanical ventilation as the first line of therapy. Nevertheless, few cases in this cohort were suitable for an awake ECMO procedure with the rationale of preventing the harmful effects of ventilation and sedation, such as ventilator-induced lung injury (VILI) and ventilator-associated pneumonia (VAP). Awake VV-ECMO, meaning ECMO without mechanical ventilation in spontaneously breathing patients, was performed in later stages. These patients were maintained awake during cannulation and throughout the ECMO run. There were no specific guidelines or indications for awake ECMO; instead, the clinical condition of selected patients, along with the clinical sense of the treating physician, determined the decision on awake ECMO. These considerations included: oxygen requirement (liter/minute), PEEP requirement (continuous positive airway pressure), inotropes requirement, and comorbidities (such as obesity, asthma, etc.). During the procedure, the sedation plan varied between patients, and local anesthesia with Lidocaine was given at the cannulation sites in all cases.

For transparency, we emphasize that some of this cohort’s data were published recently by Lorusso et al. [9]. However, the primary purpose of both studies is different.

### Statistical Analysis

Each center gathered the data of its patients into a nationwide database-REDCap^®^. The data included: patients’ demographics and characteristics; comorbidities (dyslipidemia, hypertension, diabetes, chronic kidney disease, immunosuppression, autoimmune disease, previous cerebral event, and ischemic heart disease (IHD), which was defined as previous cardiac intervention including percutaneous coronary stenting and or coronary artery bypass graft); preprocedural ventilation days; period of ECMO support; adverse events on ECMO, such as massive bleeding, cannula dislodgment events, oxygenator thrombosis and exchange, renal replacement therapy, pulmonary embolism (PE), ECMO configurational change, disseminated intravascular coagulation (DIC), and cerebrovascular accident (CVA); and outcomes (deceased, survived). Mortality was defined as in-hospital mortality. Survivors were considered patients who were weaned off ECMO and dismissed from the intensive care unit (ICU). The data were analyzed, and the patients were subsequently divided according to their outcome into the deceased group and the survivors’ group.

Data are presented as mean ± standard deviation or median and interquartile range (IQR). Continuous variables were tested with the Kolmogorov–Smirnov test for normal distribution. Categorical variables are given as frequencies and percentages. A chi-square test and Fisher’s exact test were used to compare categorical variables between the groups. Student’s t-test was performed to compare normally distributed continuous variables and the Mann–Whitney U test for non-normal distribution. The highest missing rate in any variable was 2%; therefore, no imputation was used. Multivariate Cox regression analysis was used to identify factors associated with mortality. Candidate factors appear in Table 1. Variables that were associated with one group (*p*-value < 0.05 in Table 1) were included in the regression model. In addition, we included pre-specified clinically significant variables in the model. Variables entering the premiere model were: age (as a categorical variable of >55 years of age), gender, BMI, IHD, malignancy immunosuppression, and ventilation (as a categorical variable ≤4 days prior to cannulation). We used the backward stepwise method to decide on the final model based on the likelihood ratio. The model pit was estimated by the area under the ROC curve that stands at 0.76. Statistical significance was assumed when the null hypothesis could be rejected at a *p*-value < 0.05. All *p*-values are the results of two-sided tests. Statistical analyses were conducted using R (version 3.4.1) New Jersey – USA.

## 3. Results

### 3.1. Population Characteristics

During the first COVID-19 year in Israel, 211 patients were connected to ECMO devices in the eleven involved ECMO centers. In total, 197 (93%) patients required VV-ECMO due to respiratory failure, and 14 (7%) patients required VA-ECMO support for hemodynamic instability, which were excluded from this analysis. Out of the 197 included patients, 150 (76%) were males, and 47 (24%) were females, with a mean age of 50.7 ± 12 years. The mean BMI was 32 ± 8 (kg/m^2^). Medical history of the patients included the following comorbidities: type 2 diabetes mellitus (DM) in 56 (28%), dyslipidemia in 80 (40%), hypertension in 69 (35%), chronic renal failure (CRF) in 6 (3%), ischemic heart disease (IHD) including previous percutaneous cardiac intervention (PCI) and/or coronary artery bypass grafting (CABG) surgery in 16 (8%), prior CVA or transient ischemic attack (TIA) in 5 (2%), the autoimmune condition was present in 14 (7%) of the patients and lastly, immunosuppression in 16 (8%) of the patients in the cohort. Regarding ECMO support, 17 (8.6%) patients had an awake ECMO procedure, and the rest, 180 (91.3%), were mechanically ventilated prior to cannulation for a mean duration of 3.6 ± 4.8 days. Altogether, the mean duration of ECMO support was 24.2 ± 23 days.

During the ECMO support, the following adverse events occurred: sepsis in 87 (44%), renal failure requiring renal replacement therapy with continuous veno-venous hemofiltration (CVVH) in 34 (17%), CVA in 11 (5%), PE in 4 (2%), oxygenator thrombosis requiring oxygenator exchange in 51 (25%), major bleeding in 50 (24%), disseminated intravascular coagulation (DIC) in 10 (5%), cannula dislodgment in 7 (3%), abdominal compartment syndrome in 4 (2%) and, cardiac tamponade in 2 (1%). During the study period, 10 (5%) patients required configurational change into VA ECMO due to hemodynamic instability despite significant hemodynamic support. In-hospital mortality was 106 (54%), of which 11 (10.4%) patients died in ICU after successful weaning from ECMO. In total, 91 (46%) patients were weaned-off ECMO and dismissed from the ICU. Two (1%) patients underwent successful double lung transplantations.

### 3.2. Outcomes

The deceased group was significantly older than survivors, with a mean age of 53 ± 12 years vs. 48 ± 11 years (*p* = 0.001). When age was regarded as a categorical variable of ≤/>55 years of age, there were significantly more patients over 55 years old among the deceased group (55% vs. 30% *p*-value < 0.001). No differences were found in terms of gender or BMI (Table 1).

History of ischemic heart disease (previous PCI and or CABG) was the only statistically significant comorbidity to be found at a higher rate among patients in the deceased group, with 13 (12%) patients vs. 3 (3%) in the survivors’ group (*p* = 0.034). Other comorbidities, including DM, dyslipidemia, hypertension, CRF, prior CVA/TIA, immunosuppression, and autoimmune condition, were found in similar rates among the groups. In terms of ventilation days prior to ECMO cannulation, the groups differed only when a categorical time variable of ≤/>4 days was used: 70 (77%) of the patients in the survivors’ group were ventilated for four days or less in comparison to 66 (63%) in the deceased group (*p* = 0.043). However, when a continuous time variable was tested, no differences were found between the groups (Table 1).

The rate of configurational change from VV ECMO to VA ECMO and the duration of ECMO support was similar among the groups. In terms of adverse events on ECMO, patients in the deceased group experienced more kidney failure requiring CVVH (27% vs. 4%, *p*-value < 0.001) and sepsis (51% vs. 36%, *p*-value 0.043). Rates of other adverse events on ECMO were found to be statistically insignificant, including oxygenator exchange, cannula dislodgment, CVA, PE, major bleeding, DIC, abdominal compartment syndrome, and cardiac tamponade.

**Figure 1:**
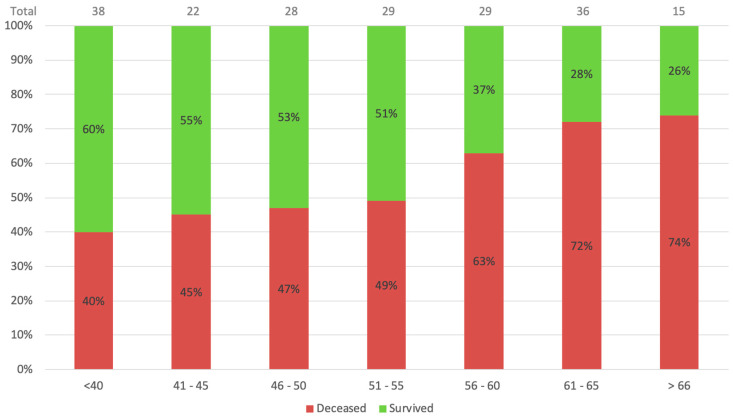
Age-related mortality. Figure 1 demonstrates the mortality and survival rates according to different age groups.

**Figure 2:**
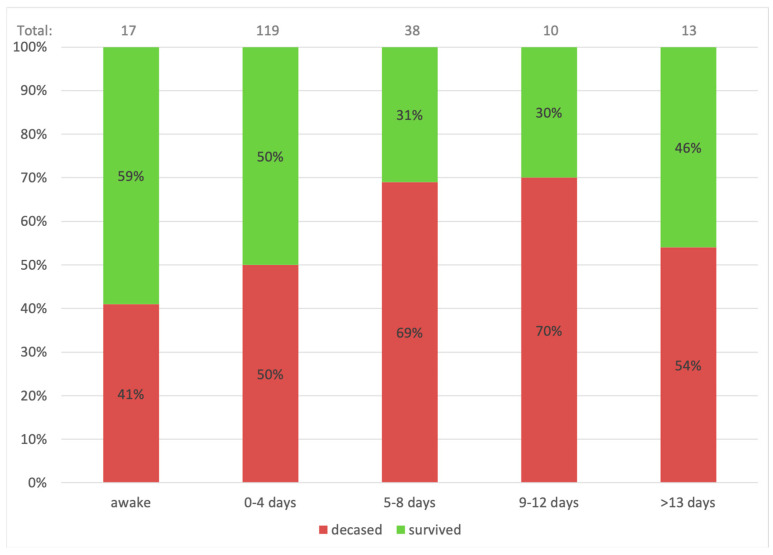
Pre-procedural ventilation days-related mortality. Figure 2 demonstrates the mortality and survival rate according to the duration of ventilation prior to connection to ECMO.

On a multivariate Cox regression analysis, candidate variables that might be associated with mortality, including variables that were associated with one group (*p*-value < 0.01 in Table 1), in addition to pre-specified clinically significant variables, were used in the model. The age and ventilation duration variables were categorized based on the clinical outcomes as presented in Figure 1 and Figure 2. With a 95% confidence interval, age over 55 (*p* = 0.005) and more prolonged ventilation (>4 days) (*p* = 0.039) were the only factors found to be associated with mortality.

## 4. Discussion

The clinical research regarding the use of ECMO in patients with COVID-19-induced respiratory failure has been increasing in recent months, including both sporadic and multi-institutional studies [3,4]. The role of ECMO in COVID-19 patients has also been addressed in the guidelines published by the World Health Organization (WHO) [10], the Centers for Disease Control and Prevention (CDC) [11], The Extracorporeal Life Support Organization (ELSO) [6] and The American Society for Artificial Internal Organs (ASAIO) [6]. Despite that, much more research is probably needed to fully comprehend the role of ECMO and its management in these complex patients. This multi-institutional retrospective study examined the vast majority of COVID-19 patients in Israel treated with VV-ECMO throughout the first pandemic year. Patients requiring VA-ECMO were excluded from the cohort. These patients needing advanced cardiac support reflect a more complex and, perhaps, critical state and vary significantly from the VV-ECMO study group.

We sought to better understand the clinical course of COVID-19 patients on VV-ECMO by comparing them according to their outcomes. The results of this study demonstrate that, compared with the deceased, the survivors were: younger, suffered less from IHD, and had shorter ventilation periods prior to ECMO cannulation. During ECMO support, the survivors had lower rates of CVVH and sepsis.

These results suggest some crucial insights on COVID-19 patients on ECMO support. First and foremost, older age (>55 years) and late cannulation (>4 days from intubation) were shown to be risk factors for mortality. Figure 1 demonstrates the age-related mortality and survival rates. It is appreciable that with each rise in age group, the mortality rate increases substantially. The mortality of patients beyond 55 years of age was over 70%, meaning fewer than 3 out of 10 patients aged over 60 years survive ECMO support. Similarly, Figure 2 shows mortality based on pre-procedural ventilation days. The highest survival is indicated for the awake ECMO group and patients ventilated for four days or less. These conclusions were also suggested by Jefferey P. et al., who showed that older age and late cannulation (12 days from diagnosis) were associated with a higher mortality rate [3]. Additionally, better survival for patients who were cannulated <7 days from ventilation was demonstrated by Raphaël Giraud et al. [12]. In this study, the difference in ventilation duration was significant when the time variable was regarded as a categorical variable of ≤4 days. Hence, performing early connection to ECMO of COVID-19 patients with overt signs of ARDS is most probably beneficial in terms of survival.

Awake ECMO, which mainly was applied during the later stages of the first pandemic year, was performed in 17 (8%) out of the 197 patients. Among patients who were connected to awake VV-ECMO, 10 (59%) patients survived, representing the highest survival in terms of pre-procedural ventilation (Figure 2). Awake ECMO seeks to avoid the harmful effects of anesthesia and mechanical ventilation, such as VAP and VILI while preserving the respiratory drive, muscular tension, and force. This strategy was applied for suitable, rapidly deteriorating patients that most probably needed ECMO support in due course and included the probability of over-supporting a few patients. This might explain the relatively higher survival rate. This study is one of the first to describe a large group of awake ECMO results, previously described only in a handful of case reports [13,14,15,16,17,18,19].

Unlike the pre-procedural ventilation days, the duration of ECMO support was not directly linked to mortality. The longest documented VV-ECMO support in this study was 139 days, for a 43-year-old male patient who was eventually successfully decannulated and is currently under a respiratory rehabilitation program. Another 56-year-old female stayed 125 days on ECMO support and was decannulated after undergoing successful double lung transplantation. Other patients with over three months of ECMO support were also successfully decannulated, although others did not survive. Altogether, there was no statistically significant difference in the duration of ECMO support between the deceased and the survivors’ groups. Thus, it could be argued that even though there is no specific timing for decannulation, prolonged VV-ECMO support is probably not a risk factor for mortality in COVID-19 patients and could be maintained for as long as required.

Prolonged ECMO support carries with it some particular concerns. Ahmadi et al. suggested that oxygenator failure is one of the risk factors for VV-ECMO failure [20]. Their study concluded that this failure is probably more abundant among COVID-19 patients due to the hypercoagulable state as a part of the hyperinflammatory immune reaction. However, a link between oxygenator exchange and mortality has not been found in this group of patients. This probably implies that a timely oxygenator exchange does not jeopardize the patient. Although oxygenator exchange may suggest a hypercoagulable state, we have learned throughout the year that continuous anticoagulation therapy is mostly, but not always, mandatory in this type of patient. During the second COVID-19 wave, the mainstay of using heparin was replaced in many centers with continuous bivalirudin treatment [21]. In addition, we have witnessed growing rates of DIC and uncontrollable bleeding in many patients, which engendered the cessation of the anticoagulative treatment while keeping ECMO on high flow. Nevertheless, major bleeding did not become a risk factor for mortality among COVID-19 patients on ECMO in Israel. This study’s PE and CVA rates were relatively low compared with similar cohorts [22,23]. This interplay between a hypercoagulable state and major bleeding is one of the most challenging aspects of these patients’ management and must always be under continual surveillance. Whether anticoagulation therapy should be maintained or ceased should be a case-to-case decision and may also be modified several times for an individual patient [24]. Further research in this field is required to clarify better our understanding and the implication of this critical aspect of ECMO support.

Additional aspects of prolonged ECMO support are acute kidney injury and sepsis. In this study, renal failure requiring renal replacement therapy with CVVH and sepsis were found in higher rates among the deceased group compared with the survivals (27% vs. 4% for CVVH, *p*-value < 0.001, and 51% vs. 36% for sepsis, *p*-value 0.043). Nevertheless, we believe that despite this, CVVH and sepsis should not be regarded as risk factors or as the causes of death. Instead, they are surrogates for critical illness and indeed, signify a worse prognosis. Inevitably, whenever required, CVVH combined ECMO therapy is effective and should be established [11,25,26]. Sepsis will sooner or later develop in many patients under prolonged ICU stay, although reports of bacteremia in COVID-19 patients on ECMO are scarce and lack statistical power [2,3,4,25]. Practicing preventive measures to avoid co-infections is an essential part of therapy, alongside treating sensitivity-specific antibiotics to avoid multi-drug-resistant bacteria.

Interestingly, we have seen more cannula dislodgment events in the early parts of the year. This could be possibly explained by the fact that ICU teams were not initially used to treating patients on prolonged ECMO support and for the other obvious reasons concerning the adjustment to the protective gear in the COVID-19 ICU settings. Jugular cannula dislodgement events occurred due to frequent patient repositioning and loosening of the cannula’s security stitches. In most cases, cannula dislodgement events necessitated cardiopulmonary resuscitation (CPR) due to a sudden drop in ECMO flow. Indeed, 85% of the patient who experienced such events did not eventually survive. The fact that cannula dislodgment events have occurred only in small numbers in survivors and non-survivors (1% vs. 6%, respectively) has probably made these events statistically insignificant (*p*-value 0.127).

### 4.1. Mortality

In a multi-institutional study by Jeffrey P. et al., 55% overall mortality was reported, including 53.7% mortality on VV-ECMO (101 of 188 patients) and 75% on VA-ECMO (9 of 12) [3]. While a systemic meta-analysis by Ramanathan et al. reported 37% mortality with VV-ECMO support [4]. This study’s overall VV-ECMO mortality was 53.8% (106 of 197). However, 10.4% (11 of 106) of these patients died in the ICU after ECMO weaning and decannulation. A similar finding was published in a systemic review by Heuts et al. [26], where 12.7% of the deceased patients died in the ICU after VV-ECMO weaning and before discharge. Similar results were recently published by Lorusso et al. [9] in large, multi-national COVID-19 ECMO patient group, where they reported 19% in-hospital mortality after ECMO weaning and 50% overall in-hospital mortality. Nevertheless, Bertini et al. [27] published an overall lower mortality rate (39%) in systematic review and meta-analysis compared with our results. Possible explanations for these differences in the mortality rates include the level of expertise for each center included in the study, the versatility in patient selection, and the time frame each study had, where at the beginning of the pandemic, higher mortality rates where documented [28].

Univariate analysis showed that older age (>55 years), presence of IHD, and cannulation beyond four days from intubation (>4 days) were the only pre-procedural variable related to increased mortality. A multivariate Cox analysis identified risk factors for mortality as older age (*p*-value 0.022) and longer ventilation duration prior to cannulation (*p*-value 0.038). IHD and other comorbidities tested in the model were not found to be risk factors for mortality on ECMO. Accounting for better survival is probably a combination of factors, including patient selection, earlier cannulation or application of awake ECMO procedures, prevention of cannula dislodgment, and possibly, the introduction of the vaccination campaign. Further extensive multi-center studies could and should be able to provide more information that will further improve the management and the outcomes of COVID-19 patients requiring ECMO support.

### 4.2. Study Limitations

This is a multi-institutional retrospective study subjected to selection bias and institutional bias. Additionally, some critical data are missing, including smoking habits, patients’ frailty status, imaging, and essential values of laboratory tests. Other missing data are on-ECMO therapies, such as adjunctive treatment strategy (e.g., steroids, antiviral drugs, convalescent plasma, interleukin-6 receptor monoclonal antibodies, prostaglandin, and hydroxychloroquine), anticoagulation trends, blood products, other co-infections, and antibiotics use. These factors might have affected the general outcomes of the patients. However, we believe that all centers have treated their patients according to the accepted international guidelines and under the direct instructions of the iECMO consultants. Therefore, we assume that the patient group is homogenous enough to be compared and published as a cohort. Lastly, out-of-hospital follow-up is not available.

## 5. Conclusions

This study adds valuable information to the forming groundwork of data and global clinical experience concerning VV-ECMO support for COVID-19 patients. It should be humbly stated that despite the growing expertise in treating COVID-19 patients, we have still not gathered enough information to overcome this complex multi-systemic disease. Based on this study, it may be argued that awake and early cannulation of younger patients may improve overall survival and a history of ischemic heart disease might indicate a reduced prognosis. Mortality for patients over 55 years of age is substantially high. In light of the recently growing number of new COVID-19 variants, it appears that more patients will need VV-ECMO for COVID-19 infection in the future.

## Figures and Tables

**Table 1:** Survival-based analysis.

	Deceased	Survivors	*p*-Value
	*n* = 106	*n* = 91	
Age (years) (mean)	53 ± 12	48 ± 11	0.003
Age > 55 years (%)	58 (55%)	27 (30%)	<0.001
Male sex (%)	84 (79%)	66 (73%)	0.175
BMI (kg/m^2^) (mean)	32 ± 8	32 ± 7	0.567
Diabetes mellitus (%)	33 (31%)	23 (25%)	0.428
Chronic kidney disease (%)	5 (5%)	1 (1%)	0.220
Hypertension (%)	38 (36%)	31 (34%)	0.881
Dyslipidemia (%)	48 (45%)	32 (35%)	0.190
Ischemic heart disease (%)	13 (12%)	3 (3%)	0.034
Malignancy/immunosuppression (%)	10 (9%)	6 (7%)	0.603
Prior CVA/TIA (%)	4 (4%)	1 (1%)	0.376
Autoimmune disease (%)	8 (8%)	6 (7%)	1.000
Ventilation days prior to cannulation (mean)	3.9 ± 4.6	3.4 ± 5.2	0.483
≤4 ventilation days prior to cannulation (%)	66 (63%)	70 (77%)	0.043
Awake VV ECMO (%)	7 (7%)	10 (11%)	0.316
Configurational change (%)	7 (7%)	3 (3%)	0.346
Days on ECMO (mean)	24 ± 22	26 ± 24	0.644
Major bleeding (%)	28 (26%)	22 (24%)	0.745
Membrane exchange (%)	24 (23%)	27 (30%)	0.328
CVVH (%)	29 (27%)	4 (4%)	<0.001
DIC (%)	6 (6%)	4 (4%)	0.755
Cannula dislodgement (%)	6 (6%)	1 (1%)	0.127
Abdominal compartment syndrome (%)	3 (3%)	1 (1%)	0.626
Cardiac tamponade (%)	2 (2%)	0 (0%)	0.500
Sepsis (%)	54 (51%)	33 (36%)	0.043
Pulmonary embolism (%)	4 (4%)	0 (0%)	0.126
CVA (%)	9 (8%)	2 (2%)	0.066

Univariate analysis of the deceased group versus the survivors’ group. The comorbidities, procedural data, adverse events on ECMO, and outcomes of each group are compared. The table demonstrates that the survivors were significantly younger, suffered less from ischemic heart diseases, and were ventilated for a shorter period prior to ECMO cannulation. This difference was proven significant only when a categorical time variable of ≤/>4 days were tested. In addition, patients in the deceased group had a higher occurrence of sepsis and acute kidney injury requiring CVVH. Data are presented as frequencies, percentages, mean, standard deviation, or median and IQR. Abbreviations: BMI—body mass index; Config—configuration; CVA—cerebral vascular accident; CVV—continuous veno-venous hemofiltration; DIC—disseminated intravascular coagulation; ECMO—extracorporeal membrane oxygenation; TIA—transient ischemic attack; VA—veno-arterial; VV—veno-venous.

## Data Availability

Data provided in this article are archived in the REDCap^®^ program (https://redcap.clalit.co.il/redcap/) (accessed on 1 February 2022) authorized and managed by the iECMOs.
